# Quantitative Evidence for the Dependence of Highly Crystalline Single Wall Carbon Nanotube Synthesis on the Growth Method

**DOI:** 10.3390/nano11123461

**Published:** 2021-12-20

**Authors:** Takashi Tsuji, Guohai Chen, Takahiro Morimoto, Yoshiki Shimizu, Jaeho Kim, Hajime Sakakita, Kenji Hata, Shunsuke Sakurai, Kazufumi Kobashi, Don N. Futaba

**Affiliations:** 1 CNT-Application Research Center, National Institute of Advanced Industrial Science and Technology (AIST), Tsukuba 305-8565, Japan; takashi.tsuji@aist.go.jp (T.T.); guohai-chen@aist.go.jp (G.C.); t-morimoto@aist.go.jp (T.M.); kenji-hata@aist.go.jp (K.H.); d-futaba@aist.go.jp (D.N.F.); 2 Nanomaterials Research Institute, National Institute of Advanced Industrial Science and Technology (AIST), Tsukuba 305-8565, Japan; shimizu.yoshiki@aist.go.jp; 3 AIST-UTokyo Advanced Operando-Measurement Technology Open Innovation Laboratory (OPERANDO-OIL), National Institute of Advanced Industrial Science and Technology (AIST), Kashiwa 227-8589, Japan; 4 Innovative Plasma Processing Group, Research Institute for Advanced Electronics and Photonics, National Institute of Advanced Industrial Science and Technology (AIST), Tsukuba 305-8568, Japan; jaeho.kim@aist.go.jp (J.K.); h.sakakita@aist.go.jp (H.S.)

**Keywords:** carbon nanotubes, crystallinity, synthetic method dependence, far-infrared spectroscopy

## Abstract

We present a study quantitatively demonstrating that the method of synthesis (gas phase, fixed bed, non-fixed bed) represents a determining factor in the level of crystallinity in growing single wall carbon nanotubes (SWCNTs). Using far infrared spectroscopy, the “effective length” (associated with the level of crystallinity) was estimated for CNTs grown using various synthetic methods (lab-produced and supplemented by commercially purchased SWCNTs) as a metric for crystallinity (i.e., defect density). Analysis of the observed “effective lengths” showed that the SWCNTs fell into two general groups: long and short (high and low crystallinity) synthesized by gas-phase methods and all other supported catalyst methods, respectively. Importantly, the “long” group exhibited effective lengths in the range of 700–2200 nm, which was greater than double that of the typical values representing the “short” group (110–490 nm). These results highlight the significant difference in crystallinity. We interpret that the difference in the crystallinity stemmed from stress concentration at the nanotube-catalyst interface during the growth process, which originated from various sources of mismatch in growth rates (e.g., vertically aligned array) as well as impact stress from contact with other substrates during fluidization or rotation. These results are consistent with well-accepted belief, but now are demonstrated quantitatively.

## 1. Introduction

Single wall carbon nanotubes (SWCNTs) have been predicted to possess exceptionally high mechanical properties as being constructed of a seamless graphitic network of sp^2^ carbon atoms, in principle. However, while 30 years have passed since their discovery [1], the properties of CNT materials have yet to reach the expectations from experimental and theoretical studies on the individual CNT level. One of the fundamental causes of this deviation is the current limitation in synthesizing perfectly crystalline SWCNTs. The presence of crystalline defects in the graphitic walls significantly degrades nearly every aspect of SWCNTs, in addition to how they assemble into a macroscopic assembly. For example, Zhu et al. demonstrated, through simulation, that the presence of a few defects in the wall of a SWCNT results in an order of magnitude drop in the strength [2]. In addition, Kondo et al. showed, through molecular-dynamics simulation, that 1% vacancy defects resulted in a 75% decrease in the thermal conductivity [3]. Furthermore, as the presence of crystalline defects affects the organization of CNTs, a corresponding decrease in macroscopic properties in such forms as yarns and films has been reported. High-performance CNT yarns and fibers have been reported using highly crystalline CNTs [4,5], but still fall far below the theoretical expectations of strength (100–200 GPa) and modulus (1–2 TPa). Tajima et al. and Dini et al. experimentally demonstrated the correlation between the decrease in electrical conductivity with an increase in the number of defects [6,7]. Furthermore, some reports have experimentally shown the influence of the SWCNT crystallinity on the thermal conductivity for vertically aligned forests or yarns [8,9,10]. Therefore, highly crystalline SWCNTs represent one of the fundamental structural aspects that impacts the properties of macroscopic CNT assemblies at the individual, mesoscopic, and macroscopic length-scales.

While the level of crystallinity is critical in harnessing the full potential of SWCNTs, the challenge is to synthesize them with little or no crystalline defects. Unlike the removal of unwanted metal catalyst or carbon materials or the sorting of specific chiral varieties [11,12,13,14], the preparation of highly crystalline SWCNTs has only been addressed at the synthesis step. Within the field of SWCNT synthesis by chemical vapor deposition (CVD) process, a number of different approaches have been advanced over the past 30 years, such as gas phase synthesis (“floating catalyst”), where the catalyst nanoparticles “float” through the reaction zone to grow isolated or aerogelated CNTs [15,16,17,18]. Further, to increase productivity and/or reaction time, supported catalyst methods, such as fixed bed [19,20,21,22,23], fluidized bed [24,25,26], and rotary kiln [27,28] processes have been developed. In these methods, powders of assembled CNTs can be grown in bulk and numerous commercial makers have been established. However, quantitatively, which method is most suited to synthesize highly crystalline SWCNTs? Conventionally, characterization of the CNT crystallinity is based on Raman spectroscopy. In short, when the amount of crystalline defects increases, the intensity ratio of graphitic (G-band) to disorder (D-band) peaks (G/D ratio) decreases [29]. Therefore, Raman G/D ratio has long been used as an indicator of CNT crystallinity. However, Raman G/D ratio is sensitive to many structural aspects of the CNT, such as carbonaceous impurities [30]. The richness of the Raman signals encompasses the various D-band peaks making the evaluation of crystallinity of CNTs elusive. In contrast, microscopic observation by transmission electron microscopy (TEM) [31] or scanning probe microscopy [32] represents a powerful technique to unmistakably locate crystalline defects at the atomic scale, but is less quantitative. For example, our recently developed gas-phase synthesis reactor produced CNTs with Raman G/D ratio of ~50 and few observable defects in TEM observation, which indicated the high crystallinity of the synthesized CNTs [33]. However, it is difficult to quantitatively compare the crystallinity with other CNTs synthesized by various kinds of methods such as arc-discharge [34], floating catalyst CVD and supported catalyst CVD.

For this study, we utilize the concept of “effective length” as a metric of crystallinity of SWCNTs, in addition to Raman spectroscopy. Morimoto et al. reported a method for estimating the crystallinity of CNTs by measuring its “effective” length by one-dimensional plasmon resonance from Fourier-transform infrared spectroscopy in the far infrared region, which we denote here as FIR spectroscopy [35,36,37]. In contrast to the physical length, which is the measured length of the CNT, the effective length is defined as average length segment between defects as estimated from the FIR absorption peak using a one-dimensional plasmon model. Because the effective length is less sensitive to the diameter and carbonaceous impurities, such as amorphous and graphitic carbon, which interfere with the evaluation of CNT crystallinity by Raman spectroscopy, effective length can act as a quantitative indicator for the CNT crystallinity. This approach provides a simple, reliable, and quantitative method for crystallinity characterization (i.e., defect density).

Here, we report quantitative evidence, using both Raman and FIR, that the method of synthesis (gas phase, fixed bed, non-fixed bed) represents a determining factor in the level of crystallinity (“effective length”) for the large-scale synthesis of CNTs. We synthesized CNTs in methods spanning these three chemical vapor deposition approaches and examined each spectroscopically. Analysis of the observed “effective lengths” revealed that the CNTs fell into two general groups: (1) longer (i.e., higher crystallinity), which consisted of gas phase-produced CNTs, and (2) shorter (i.e., lower crystallinity), which consisted of supported catalyst-produced CNTs. We further examined numerous commercially available CNTs to support the generality of our results. The effective length difference was stark with the longer group ranging from 700 to 2200 nm, and the shorter group ranging from 110 to 490 nm. We interpret that the difference in the crystallinity stems from the stress at the nanotube-catalyst interface felt during the growth process, which originates from the presence of adjacently bundled CNTs as well as impact stress from contact with other substrates during fluidization or slumping. These results show quantitative evidence that suggests that high yield synthesis techniques are intrinsically limited in the level of crystallinity.

## 2. Materials and Methods

### 2.1. Materials

CNTs (eDIPS (EC2.0, EC1.5), TUBALL, Meijo Arc (APJ-SSA), SGCNT (ZEONANO SG101), CoMoCAT (CG100, CG200, FW100)) were purchased or received from Meijo Nano Carbon Co., Ltd. (Aichi, Japan), OCSiAl, Meijo Nano Carbon Co., Ltd (Aichi, Japan)., Zeon Corporation (Tokyo, Japan), CHASM Advanced Materials, Inc (Canton, MA, USA), respectively.

### 2.2. Synthesis of CNTs by Plasma-Assisted Gas-Phase Process

CNTs were synthesized in a horizontal quartz reactor using microplasma-produced Fe nanoparticles as catalyst and C_2_H_4_ as carbon feedstocks [33]. For the production of Fe nanoparticles, Fe(CO)_5_ (0.06 vol%) in a CO_2_ (0.25 vol%)/H_2_ (3 vol%)/Ar (200 sccm) mixed carrier gas was passed through the microplasma generated inside an alumina capillary (inner diameter: 1.5 mm) by applying ultrahigh frequency power (450 MHz, 30 W) to the coiled electrode surrounding the capillary [38,39]. The quartz reactor consists of a coaxial double tube (inner diameter of the inner tube: 15 mm) and an electric furnace (set temperature: 925 °C, heating zone length: 150 mm). C_2_H_4_ (3 vol%) in N_2_ (500 sccm) was preheated by passing the outer tube and then mixed with microplasma-produced Fe nanoparticles to nucleate and grow CNTs in the inner tube. Grown CNTs were collected by a PTFE filter (Merck Japan (Tokyo, Japan); JCWP04700) placed at the exhaust section of the reactor.

### 2.3. Synthesis of CNTs by Fixed Bed

CNTs were synthesized from catalyst nanoparticles on a fixed bed in a furnace reactor. For the preparation of standard catalyst (Fe/Al_2_O_3_), Al_2_O_3_ (40 nm) and Fe (1.8 nm) were sequentially deposited by sputtering onto silicon wafer with thermally grown oxide layer [40]. The triple-layered catalyst (Al_2_O_3_/Fe/Al_2_O_3_) was also prepared by sequential deposition of Al (5 nm) followed by an oxygen plasma treatment (3 min at 300 W), Fe (1.0 nm), and Al_2_O_3_ (0.5 nm) for bottom, middle and top layers, respectively, [41]. The standard catalyst was reduced at 750 °C for 6 min using 90 vol% H_2_ in N_2_ (total flow: 1000 sccm). Then, CNTs were synthesized by water-assisted CVD method (“Super-growth” CVD method) at 750 °C using 0.4 vol% C_2_H_2_ as a carbon feedstock, H_2_O as a growth enhancer and N_2_ as a carrier gas (total flow: 1000 sccm). The CNT synthesized by “Super-growth” CVD method using the standard catalyst is hereafter denoted as SG (std). To synthesize tall, higher crystalline CNT forest, the triple-layered catalyst was reduced at 650 °C for 3 min using 90 vol% H_2_ in N_2_ (total flow: 1000 sccm). Then, CNTs were synthesized by “Super-growth” CVD method at 750 °C using 1 vol% C_2_H_4_ as a carbon feedstock, H_2_O as a growth enhancer and N_2_ as a carrier gas (total flow: 1000 sccm). The CNT synthesized by “Super-growth” CVD method using the triple-layered catalyst is hereafter denoted as SG (triple layer).

### 2.4. Synthesis of CNTs by Non-Fixed Bed (Rotary Kiln)

CNTs were synthesized from catalyst nanoparticles on a non-fixed bed in a furnace reactor. Catalyst was prepared by deposition of Al_2_O_3_ and Fe on ZrO_2_ beads (diameter: 0.65 mm). These beads were put in a cylindrical metallic tube (inner diameter: 22 mm, length: 150 mm) with mesh caps on both ends to allow gas flow. The container was placed in a 1-inch quartz tube reactor and rotated with the length direction as the rotation axis (3 rpm). The catalysts were reduced at 750 °C for 18 min using 90 vol% H_2_ in N_2_ (total flow: 2000 sccm). Then, CNTs were synthesized by “Super-growth” CVD method at 750 °C using 20 vol% C_2_H_4_ as a carbon feedstock, H_2_O as a growth enhancer and N_2_ as a carrier gas (total flow: 1000 sccm).

### 2.5. Characterization of CNTs

The synthesized CNTs were characterized by scanning electron microscopy (SEM; Hitachi, S-4800), Raman spectroscopy (Thermo Fisher Scientific (Tokyo, Japan), DXR3) with 532 nm excitation wavelength, and transmission electron microscopy (TEM; TOPCON (Tokyo, Japan), EM-002B). The FIR spectra were measured using a Fourier-transform infrared spectrometer (Bruker (Kanagawa, Japan), Vertex 80v) in the 70–8000 cm^−1^ range and a terahertz time-domain spectroscopy system (Otsuka Electronics (Osaka, Japan), TR-1000) in the 5–70 cm^−1^ range. For FIR measurement, a CNT thin film was fabricated by vacuum filtration of CNT dispersion in water with 1 wt% sodium dodecylbenzene sulfonate (SDBS) prepared by sonication for 1 min and transferred onto a high-resistance silicon substrate. Effective length of CNT was estimated from the peak position of FIR absorption as shown in references [6,35,36].

## 3. Results and Discussion

**Figure 1:**
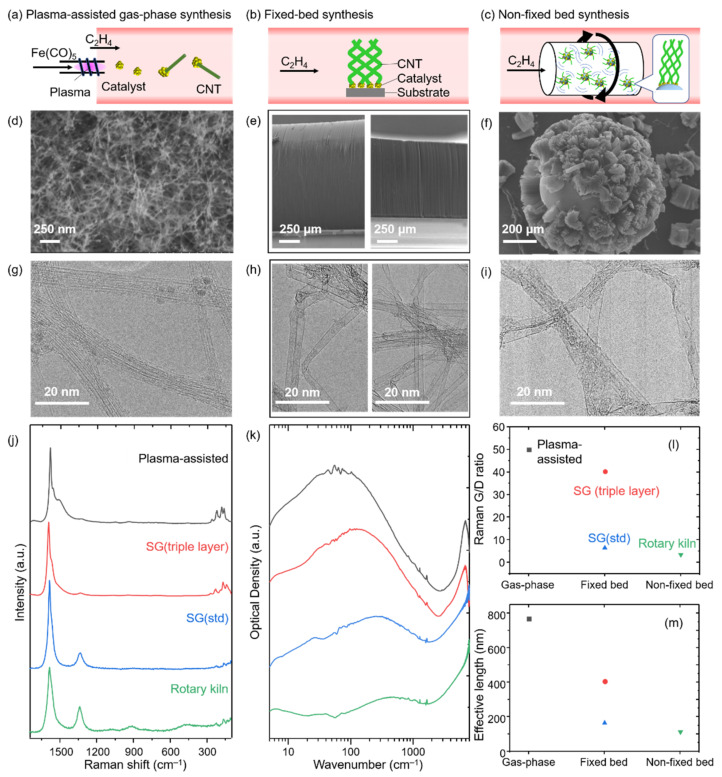
(**a**–**c**) Schematic illustrations of CNT synthesis processes. (**d**–**f**) SEM and (**g**–**i**) TEM images of the products produced by plasma-assisted gas-phase, fixed bed (triple-layer catalyst and standard catalyst) and non-fixed bed (rotary kiln) processes, respectively. (**j**) Raman and (**k**) FIR spectra of plasma-assisted gas-phase synthesized CNTs (black), SG (triple layer) (red), SG (std) (blue), and rotary kiln synthesized CNTs (green). Plots for (**l**) Raman G/D ratio and (**m**) effective length.

We began by examining a set of SWCNTs synthesized by the three general CVD techniques: gas-phase process (using a plasma-assisted approach), fixed bed process (using two kinds of supported catalysts), and non-fixed bed process (rotary kiln). Each of the grown CNT varieties was then characterized by FIR and Raman spectroscopies to evaluate the level of crystallinity (effective lengths and G/D ratios) (Figure 1). Each method is described in detail in the Materials and Methods section.

The gas-phase synthesis process yielded small diameter CNTs, which were collected onto a PTFE filter placed at the exhaust section of the reactor (i.e., dead-end filtration). SEM observation of the CNT film showed the straight fibrous materials (Figure 1d). TEM observation of the products confirmed the production of small diameter (average: 1.2 nm) and non-kinked SWCNTs, which suggests few defects in CNT graphitic walls (Figure 1g). Further spectroscopic characterization showed more quantitative evidence for the relatively high level of crystallinity. Specifically, the Raman G/D ratio was estimated to be ~50 using a 532 nm excitation wavelength laser (Figure 1j,l). In addition, FIR spectroscopy revealed a primary absorption peak at ~60 cm^−1^ (Figure 1k), which corresponded to an effective length of ~770 nm (Figure 1m).

For the two varieties of the fixed bed synthesis, the height/diameter was found to be ~1200 μm/~3 nm and~1000 μm/~2 nm for SG (std) and SG (triple layer), respectively (Figure 1e,h). As reported in numerous articles, the height and vertical alignment of this fixed bed method represents one of its primary unique features based on the self-assembly of CNTs through the crowding effect [42,43]. Further spectroscopic characterization showed contrasting levels of crystallinity of the synthesized SWCNTs. Specifically, the Raman G/D ratios for SG (std) and SG (triple layer) were estimated to be ~6.5 and ~40, respectively (Figure 1j,l). Furthermore, effective length analysis resulted in values of 167 and 450 nm, respectively (Figure 1k,m).

The rotary kiln synthesized CNTs resulted in vertically oriented arrays of SWCNTs from bead substrates with a height of ~200 μm and a diameter of ~3 nm (Figure 1f,i). As expected from this high yield approach, the volumetric yield was estimated to be ~15-times higher than that of the fixed bed approach. The specific surface area for these samples using the Brunauer-Emmett-Teller (BET) formulation from nitrogen adsorption/desorption isotherms was estimated to be about 1100 m^2^/g, which indicates the product was composed mostly of SWCNTs with high purity [44]. Interestingly, the Raman G/D ratio for these SWCNTs was estimated to be only ~3.5, and the effective length analysis resulted in 113 nm (Figure 1j–m).

To compare the grown CNTs, the Raman spectra, FIR spectra, G/D ratios, and effective lengths were plotted in descending order (Figure 1j,k,l,m, respectively). From these plots, we can make several observations. First, the Raman spectra for the series of CNTs shows a clear G-band (Figure 1j). However, the spectra of the grown CNTs for the three methods begin to differ where the G-band exhibits different levels of broadening, and the D-band increases in relative intensity. Two, the plot of the calculated G/D ratios shows two distinct groups: one at >40 and another at <10 (Figure 1l). The Raman data suggests that a difference in the number of defects present in the CNTs based on the synthesis method may be detectable. Third, overlaid plots of the FIR spectra show a generally similar trend as observed in the Raman spectra, yet with the peak locations differing based on synthetic method (Figure 1k). Specifically, the absorption peaks for each type of CNTs occurred at ~60, ~130, ~280, and ~480 cm^−1^, which corresponds to effective lengths of 770, 407, 167, and 113 nm, respectively. Fourth, the plot of the effective lengths shows a similar tendency as that of the Raman G/D ratio with the exception that the triple-layered catalyst grown CNTs appear more removed from the gas-phase CNTs and closer to the groups of fixed bed and non-fixed bed CNTs (Figure 1m). The mismatch between the G/D ratio and the FIR measurements highlights the sensitivity of Raman spectroscopy to multiple structural features, including carbon impurities, which can affect its general use for crystallinity assessment. In contrast, the FIR absorption peak is primarily derived from the one dimensional-plasmon resonance of SWCNTs and not carbon impurities, which affords accurate and quantitative CNT crystallinity evaluation. Taken together, these results suggest a correlation between the synthetic method and the level of crystallinity.

**Figure 2:**
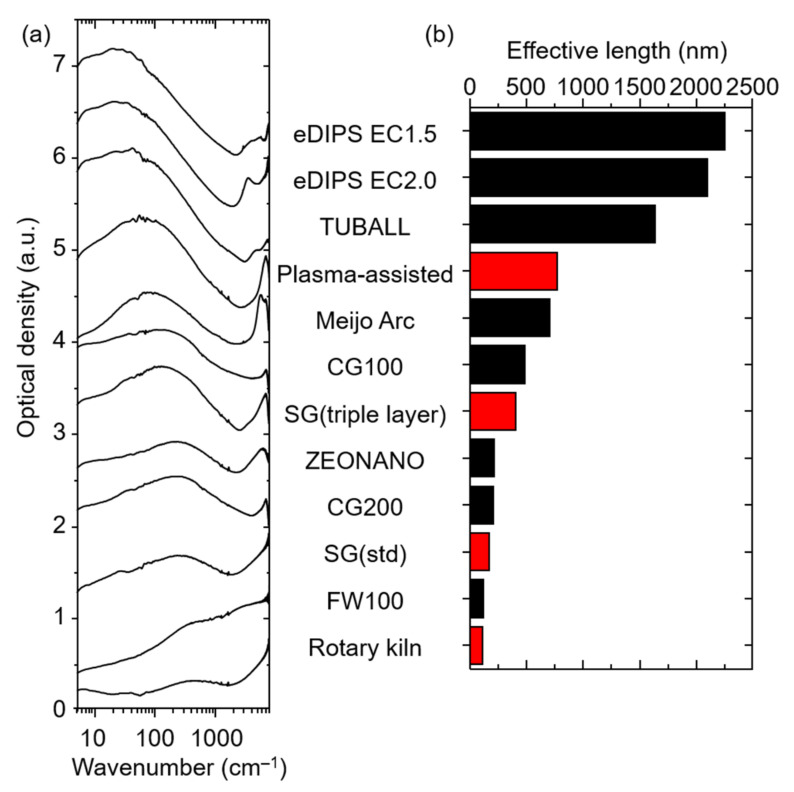
(**a**) FIR absorption spectra of various kinds of CNTs and (**b**) their effective lengths estimated from the absorption spectra.

With our current set of data limited to four data points, we sought to assess the generality of the observations, by performing the identical examination of numerous commercially available CNTs (eDIPS (EC1.5, 2.0), TUBALL, Meijo Arc APJ-SSA, ZEONANO SG101, CoMoCAT (CG100, CG200, FW100)). As previously reported, CNTs having the longer effective length showed absorption at lower wavenumber [37]. As a set, the absorption spectra showed peaks spanning ~20 cm^−1^ to as high as ~200 cm^−1^ (Figure 2a). Again, the effective lengths were estimated from the peak location and included in the plot of Figure 2b in descending order. From this plot we make several observations. First, the trend of the set of our synthesized CNTs agrees well with this larger set of commercial CNTs. Second, the effective lengths widely varied among the different makers from as high as 2200 nm down to ~100 nm. Third, two makers, specifically, stood separately from the rest of the set of SWCNTs with effective lengths estimated at ~2200 and 1700 nm, which was about double that of the next highest. Fourth, besides a slight middle region at 600–700 nm, all other CNTs fell in the range of 100 to 450 nm. Finally, comparing our synthesized CNTs, the plasma-assisted gas-phase synthesis was similar to that of Meijo Arc (705 nm), the vertically aligned fixed bed CNT (SG (triple layer)) was 407 nm, and the rotary kiln synthesized CNT fell within the lowest grouping (113 nm).

**Figure 3:**
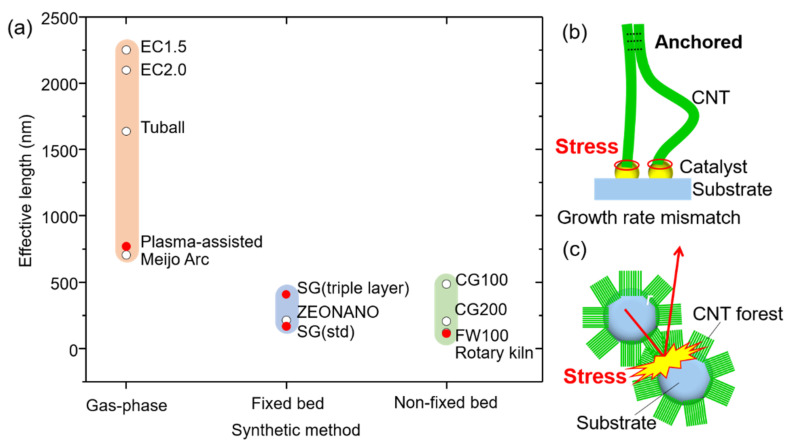
(**a**) Effective lengths of CNTs classified by synthesis methods. Schematic illustrations showing (**b**) the stress loaded to the catalyst-CNT interface by the growth rate mismatch between CNTs and (**c**) additional stress by collision between substrates during CNT growth.

This set of “effective length” data provided us with the unique opportunity to examine the relationship further, quantitatively, between crystallinity and synthetic method. We replotted the data as a function of synthetic method, and a clear trend emerged (Figure 3a). Within the limited set of SWCNTs, we see that the gas-phase synthesized SWCNTs possessed the longest effective length (700–2250 nm), followed by the vertically aligned fixed bed SWCNTs (170–410 nm), and non-fixed bed SWCNTs (110–490 nm). This result suggested a synthesis-dependence on the crystallinity. To further investigate this point, we classified the CNT samples as follows: the first group was denoted as gas-phase synthesis (eDIPS (EC 1.5, EC 2.0), TUBALL, Meijo Arc) because CNTs are grown from isolated metal catalyst nanoparticles in gas phase. The second group was denoted as fixed bed-supported catalyst (SG (std), SG (triple layer), SG101), because the CNTs are grown from catalyst nanoparticles supported on a non-interacting substrate. The final group, denoted as rotary kiln/fluidized bed, included CoMoCAT (CG100, CG200, FW100) because CNTs were grown from the catalyst nanoparticles supported on substates in a non-fixed bed system. From this plot, several interesting features are evident. First, the results generally agreed with the trend observed with the lab-grown CNTs made by three different synthetic methods. Second, a clear distinction in the crystallinity (effective length) emerged among the synthesis methods. CNTs grown from gas-phase synthesis exhibited the longest effective length (highest crystallinity) spanning from 700 to 2250 nm. This agrees with well-accepted belief but had been only shown with Raman spectroscopy. (Note: In the case of the 700 nm effective length CNTs, we interpret that the effective length is limited by the physical length (1500 nm). Therefore, intrinsically, the effective length is expected to be longer.) Meanwhile, CNTs grown on supported catalysts showed significantly lower effective lengths spanning 110–490 nm. It is noteworthy that while other synthetic factors, such as process temperature and carbon feeding level (i.e., growth rate), are known to also affect crystallinity, we interpret that these synthetic factors contribute to the observed variance within each of the synthetic methods. Our results quantitatively indicate that despite the variances resulting from growth conditions, the growth method fundamentally limits the level of crystallinity.

Our examination of the general processes involved in synthesis method strongly suggests that stress concentration (force) at the catalyst-nanotube interface, unique to each method, is the basis behind our observed experimental findings. As the interface between the catalyst nanoparticle and the growing CNT consists of only a thin ring, it is likely that the delicate nature of this interface can play a significant role in the structure of the CNTs.

We now examine possible stress factors, which are intrinsic to each synthetic method. For gas-phase synthesis, individual catalysts “float” through the growth reactor in a carbon-rich gas flow during which time the CNTs precipitate. As neither the catalysts nor the CNTs are bound, minimal external stress is added to the “growth interface”, in principle. In fact, even in the case where self-assembly and aerogelation occur, which would anchor the CNTs, the catalyst remains unbound. Wei et al. demonstrated this aspect in synthesizing structurally perfect CNTs by a “kite-growth” mode, where the catalyst could float in the gas flow while the CNT tip was fixed to a substrate. In this way, they demonstrated 50 cm long CNTs, possessing exceptionally high crystallinity as demonstrated by their vibrational properties [45]. Hence, in principle, as gas-phase synthesis presents the lowest possibility of inducing stress at the growth interface, this agrees with our experimental results, which showed that this method provides the highest level of crystallinity among the CVD processes.

In contrast, vertically aligned fixed bed and non-fixed bed methods are based on using arrays of catalysts supported on substrates and encounter stress both from the anchored catalysts and the CNT assembly. The van der Waals-induced interfacial shear strength between CNTs is sufficient to resist slip during CNT growth and has been estimated between 0.05 and 0.25 GPa [46]. For typical supported catalysts on flat substrates, CNTs grow from catalyst deposited onto an alumina-coated substrate and are, therefore, anchored to the substrate [47]. As the CNTs initiate growth, the very principle that affords vertical alignment, e.g., the self-assembly through the crowding effects of adjacent CNTs, forms a “crust”, which now anchors the tops of the CNTs to each other. Hence, the individual catalyst-CNT pairs are now anchored at both ends (Figure 3b). Numerous factors are sources of mechanical stress, such as mismatch in growth rates in adjacent catalysts caused from variations in catalyst diameter and carbon feeding. The strength of adhesion-induced coupling between CNTs, along with diameter-dependent difference in the growth rate, has been reported to impart a load of ~10 nN on a catalyst particle during growth [46,48]. Mesoscopically, the results of this phenomenon have been well observed and characterized by the mixture of aligned and “wavy” CNTs particularly for multiwall CNT forests [49,50]. As the entire surface of CNTs has formed a crust, the vertical growth progression must occur as a unit; differences in catalyst density represent another possible source of stress, which further exacerbates this issue. It is well-known that the diameter distribution for vertically aligned CNTs is exceptionally broad [47]. In addition, variation in particle density has been observed for substrate as small as 10–20 mm. To demonstrate this point, we carried out the high-resolution SEM examination on 16 regions on a 20 × 20 mm substrate following the formation of nanoparticles and estimated the particle density. The average value was found to be 6.44 × 10^3^ particles/μm^2^, which is typical for SWCNT arrays (Figure S1). However, a 30% variance in particle density was also observed, which is expected to affect the growth rates. These mismatches in growth rates can induce compressive and tensile stress to the catalyst-CNT interface [51]. Hart et al. reported that CNT synthesis under stress will result in a reduction in alignment as well as reduction in crystallinity [52]. For non-fixed bed methods, such as rotary kiln or fluidized bed, if the particle density is sufficient, then only the CNTs are subjected to the same assembly-driven stress as fixed bed methods, but also external stress generated through constant contact from the “slumping” or “fluidization” of catalyst-coated particles as well as growth under externally applied weight (Figure 3c). Our effective length results support this concept that collision induced stress on the CNTs growing in non-fixed bed methods contribute to the defect formation on the CNTs. Whatever the source of the stress, previous reports have demonstrated that mechanical force on the catalyst/CNT interfaces leads to creating defects and bends in CNTs to accommodate the applied forces [46].

## 4. Conclusions

In conclusion, we have quantitatively demonstrated the significant effect of synthetic method on the level of CNT crystallinity. Using both Raman and FIR spectroscopy, we have observed a clear correlation between the synthetic method (gas-phase, fixed bed, and non-fixed bed) and the level of crystallinity. Using FIR spectroscopy, the effective length of each type could be quantified, which revealed that the gas-phase synthesis clearly generated CNTs of the highest crystallinity among various CVD methods (effective lengths as high as 2200 nm). In contrast, supported growth methods, by fixed bed and non-fixed bed (rotary kiln/fluidized) methods resulted in significantly lower crystalline quality (effective lengths of ~110–490 nm). We interpret that this observed difference stems from the stress imparted at the catalyst-CNT interface, which is inherent to each method. As high levels of crystallinity are necessary for high performance applications, these results provide guidance on which synthesis methods are best suited to reach these goals. Furthermore, these results show quantitative evidence suggesting that high-yield synthesis techniques are intrinsically limited in the level of crystallinity.

## Data Availability

Not applicable.

## Supplementary Materials

The following are available online at https://www.mdpi.com/article/10.3390/nano11123461/s1, Figure S1: Spatial distribution of number density of nanoparticles (NPs) on the 20 mm × 20 mm substrate measured by SEM images of the catalysts.
