# Supplementary material for: Quantitative Evidence for the Dependence of Highly Crystalline Single Wall Carbon Nanotube Synthesis on the Growth Method
Source: Nanomaterials (Basel). 2021 Dec 20;11(12):3461. doi: 10.3390/nano11123461 (PMC8706310; doi:10.3390/nano11123461)
Supplement: Supplementary file 1 [file nanomaterials-11-03461-s001.zip › nanomaterials-1476425-supplementary.pdf]

nanomaterials

*Supplementary Materials*

# Quantitative Evidence for the Dependence of Highly Crystalline Single Wall Carbon Nanotube Synthesis on the Growth Method

**Takashi Tsuji [1,†], Guohai Chen [1,†], Takahiro Morimoto [1], Yoshiki Shimizu [2,3], Jaeho Kim [4], Hajime Sakakita [4], Kenji Hata [1], Shunsuke Sakurai [1,*], Kazufumi Kobashi [1,*] and Don N. Futaba [1]**

1 CNT-Application Research Center, National Institute of Advanced Industrial Science and Technology (AIST), Tsukuba 305-8565, Japan; takashi.tsuji@aist.go.jp (T.T.); guohai-chen@aist.go.jp (G.C.); t-morimoto@aist.go.jp (T.M.); kenji-hata@aist.go.jp (K.H.); d-futaba@aist.go.jp (D.N.F.)
2 Nanomaterials Research Institute, National Institute of Advanced Industrial Science and Technology (AIST), Tsukuba 305-8565, Japan; shimizu.yoshiki@aist.go.jp
3 AIST-UTokyo Advanced Operando-Measurement Technology Open Innovation Laboratory (OPERANDO-OIL), National Institute of Advanced Industrial Science and Technology (AIST), Kashiwa 227-8589, Japan
4 Innovative Plasma Processing Group, Research Institute for Advanced Electronics and Photonics, National Institute of Advanced Industrial Science and Technology (AIST), Tsukuba 305-8568, Japan; jaeho.kim@aist.go.jp (J.K.); h.sakakita@aist.go.jp (H.S.)
* Correspondence: shunsuke-sakurai@aist.go.jp (S.S.); kobashi-kazufumi@aist.go.jp (K.K.)
† These authors contributed equally to this work.

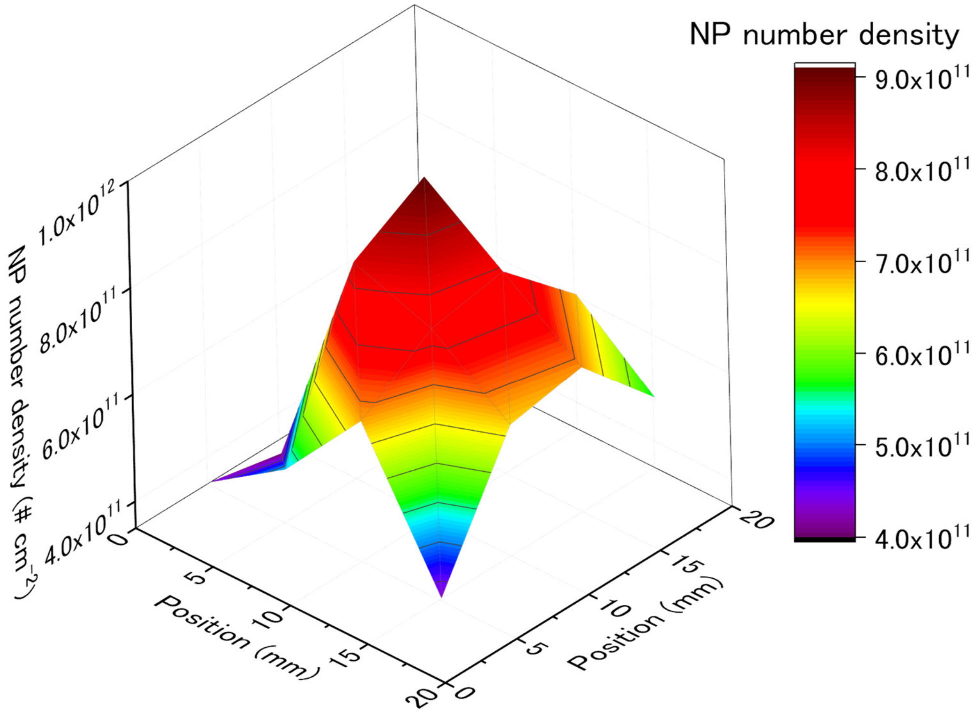


**Figure S1.** Spatial distribution of number density of nanoparticles (NPs) on the 20 mm × 20 mm substrate measured by SEM images of the catalysts.
